# Seminal Contributions of Timothy J. Crow

**DOI:** 10.1017/S0033291725000182

**Published:** 2025-03-07

**Authors:** Lena Palaniyappan, Peter F. Liddle

**Affiliations:** 1 Douglas Mental Health University Institute, Department of Psychiatry, McGill University, QC, Canada; 2 Department of Psychiatry, Schulich School of Medicine & Dentistry, University of Western Ontario London, ON, Canada; 3 Robarts Research Institute & Lawson Health Research Institute, London, ON, Canada; 4 Institute of Mental Health, University of Nottingham, Nottingham, UK

**Keywords:** psychosis, continuum, evolution, pathophysiology, subtypes, asymmetry, language, sex

## Abstract

We recall the life and work of Timothy J. Crow, whose contributions provided great insights into the pathophysiology of schizophrenia and continue to shape many questions in the field. We compile his key works relating to psychotic disorders, focusing on the trajectory of his theoretical stance. Our account is interlaced with our own interpretation of the evidence that influenced Crow’s arguments over the years as well as his scientific method. Crow has had a significant impact on the neuroscience of schizophrenia. Many of his observations are still valid and several questions he raised remain unanswered to date.

## 1 Introduction

Crow’s contributions have profoundly shaped our pursuit of schizophrenia’s pathophysiology. He pioneered the first neuroimaging study revealing ventricular abnormalities, refined the notion of continuum of psychosis, examined and dismissed the viral hypothesis of schizophrenia, and developed an early subtyping schema that preceded current investigations on the heterogeneity of schizophrenia. His work on genetics, cerebral asymmetry and language, though unfinished in his lifetime, exemplifies his approach to the schizophrenia problem. Here, we trace his 5 major contributions in a chronological order. We elucidate how a remarkable psychiatric scientist rigorously pursued clinical theory building and testing at a time of rapid technological growth in neuroscience.

## 2 Dopamine hypothesis and the mechanism of action of antipsychotic medication

As a result of his pharmacological and behavioural studies of self-stimulation in rats, performed during his PhD, Crow played a cardinal role in establishing the role of dopamine in incentive motivation (Crow, 1973). Subsequently Seeman and colleagues (Seeman et al., 1976) demonstrated that all effective antipsychotic drugs available at that time blocked dopamine receptors at concentrations that were correlated with their clinical potency, adding to the speculation that dopamine blockade was a crucial aspect of antipsychotic activity. Crow and his colleagues at Northwick Park hospital (Johnstone et al., 1978), carried out a trial comparing the effects of the alpha isomer of flupentixol, which binds to dopamine receptors, with the effects of the beta isomer, which does not bind to that receptor despite sharing many of the other pharmacological actions of the alpha isomer. They demonstrated that the antipsychotic action of flupentixol is confined to the alpha-isomer. Furthermore, the therapeutic effects were largely confined to the positive symptoms: delusions, hallucination and formal thought disorder. For the subsequent four decades, the hypothesis of dopaminergic overactivity has played a major role in accounts of the neurochemistry of schizophrenia. Recent studies using Positron Emission Tomography indicate an excess of dopamine in presynaptic nerve terminals in schizophrenia, though a similar presynaptic dopamine excess is also observed in psychotic bipolar disorder (Jauhar et al., 2017) consistent with the evidence from the flupentixol study that dopamine blockade is effective against positive psychotic symptoms, rather than acting on the on a process that is central to the entire gamut of symptoms of schizophrenia.

## 3 Cognition and brain structure in schizophrenia

Crow was fascinated by the degree of cognitive impairment observed in cases of chronic schizophrenia, most notably the age disorientation exhibited by an appreciable proportion of long-stay patients who had a fixed belief that their age was only a few years greater that the age at time of admission to hospital, despite the fact that this was in many cases several decades in the past (Crow and Mitchell, 1976). Many cases exhibited impairment on the cognitive tests comparable to that observed in neurological cases with overt brain damage. The severity of the observed cognitive impairments led him to challenge the prevailing assumption that such impairments were not associated with overt brain damage. The development of Computed Tomography scanning in the mid-1970’s provided the tool to address the issue. In the landmark paper (Johnstone, et al., 1976), Crow and his colleagues at Northwick Park hospital reported that in a sample of severely impaired long-stay patients from Shenley Hospital, the cerebral ventricles were enlarged relative to overall brain size. They reported that this group of severely impaired patients also exhibited marked cognitive impairments (including poor performance on serial sevens and delayed recall) that were significantly correlated with the degree of ventricular enlargement.

In the subsequent years the findings of other investigators provided conflicting evidence regarding ventricular enlargement in schizophrenia. More recent meta-analysis confirms the early reports from Crow and colleagues, but the effect is smaller than initially reported (Sayo et al., 2012). Numerous subsequent investigations have also confirmed impairment of many aspects of cognition in schizophrenia (McCutcheon et al., 2023), with diverse associations between the cognitive impairments and abnormalities of brain structure and function in schizophrenia. In their review, McCutcheon and colleagues (2023) propose that these abnormalities converge on a common mechanism entailing imbalanced interactions between excitatory and inhibitory (E/I) neurons of cortical microcircuits.

## 4 Positive and negative symptoms and subtypes of schizophrenia

The finding of the flupentixol study, together with the observation that abuse of amphetamine, a drug that promotes the release of dopamine from presynaptic terminals can produce an illness strongly resembling paranoid schizophrenia, added to the growing evidence that dopamine plays a crucial role in acute psychosis. However, other evidence, most notably the finding of cerebral ventricular enlargement and marked cognitive impairment in individuals experiencing severely disabling chronic schizophrenia (Johnstone et al., 1976), suggested to Crow that in least some cases, there is a fundamental deficit distinct from that pathophysiological process underlying acute schizophrenic disturbance (Crow, 1980). In this groundbreaking paper, he proposed that two distinguishable pathological processes might occur in schizophrenia. The first (which he labelled the type I syndrome) is characterised by the positive symptoms (delusions, hallucinations, and formal thought disorder) and is associated with abnormal dopaminergic transmission; the second (the type II syndrome) is characterised by negative symptoms (affective flattening and poverty of speech) unrelated to dopaminergic transmission but likely associated with intellectual impairment and structural brain changes.

Recent factor analyses of the symptoms of schizophrenia embracing a more diverse range of symptoms reveal at least five distinguishable symptom clusters (Shafer & Dazzi, 2019), likely reflecting five distinguishable pathological processes. With sophisticated molecular imaging approaches, evidence has accrued in favour of a subgroup with predominantly dopaminergic aberrations, though uncertainty around the longitudinal stability of this subgroup still remains (Howes et al., 2025). A more stable subgroup with structural changes (reduced cortical grey matter) has also been consistently observed, though its association with cognitive impairment and negative symptoms has been less remarkable (Liang et al., 2024).

Crow’s concept of distinguishable pathological processes leading to distinct clusters of symptoms provided a fruitful foundation for subsequent investigations. However, in later years Crow himself focussed strongly on identifying a single pathological process at the heart of the illness, as we discuss below.

## 5 Viral transmission and continuum hypothesis

Preliminary evidence indicative of virus-like particles in the CSF of a minority of patients with schizophrenia (Crow et al., 1979; Tyrrell et al., 1979) led Crow to speculate that schizophrenia was a viral illness. Subsequently, he observed that pairs of siblings concordant for schizophrenia had similar age of onset, but not similar calendar time of onset (Crow, 1984; Crow & Done, 1986). This lack of evidence for a contagion hypothesis or horizontal transmission between siblings led him to propose a ‘provirus’ acquired either by prenatal infection or in the germ-line from an affected parent, might be integrated in the genome leading to schizophrenia (Crow, 1984). He speculated that this virus (possibly a retrovirus) might interact with a proto-oncogene (a gene that promotes cell growth) that normally induces the asymmetrical brain growth and cerebral dominance. This proposed interaction between provirus and proto-oncogene would interfere with the development of normal laterality at a specific developmental stage, leading to disturbed asymmetry in schizophrenia, and consequent disturbance of the capacity for communication and social interaction.

Crow postulated that evolutionary pressures lead to a high degree of variability in the postulated interaction between the provirus and proto-oncogene (Crow, 1987). He proposed that a spectrum of severity of the distributed interaction between the provirus and proto-oncogene might generate a continuum of severity of psychotic illness extending from unipolar through bipolar affective and schizoaffective disorder to schizophrenia.

## 6 “Psychosis is the price we pay for language”

This idea has remained one of Crow’s longest-held theories of origin of the continuum of psychosis. The earliest mention of the relevance of language appeared in his works in 1984 in the context of viral theory. Discussing the post-mortem findings of asymmetry in schizophrenia from the Runwell Hospital collection (see (Kasper et al., 2010) for more context) and left-lateralized increase in limbic dopamine (Reynolds, 1983), Crow stated that hemispheric asymmetry “.. is an unusual and specific evolutionary development, associated with inter-individual communication” (Crow, 1984). He developed this idea further in his later thesis on pseudoautosomal locus for schizophrenia genes (Crow, 1988). Since then, in almost all of his subsequent papers, language assumed a central place in his arguments on the function of psychosis-related genetic changes (Crow, 1995).

Besides the neuroanatomical feature of disrupted asymmetry in schizophrenia, Crow considered 2 epidemiological features as critical explananda for pathogenetic theories of schizophrenia (Crow, 1997): (1) the nuclear syndrome of schizophrenia defined by its ‘first rank’ symptoms are universally present in all populations, across time, with approximately the same incidence despite its reproductive disadvantage (2) females have consistently later onset of schizophrenia than males. From this, he deduced that the genetic factors of schizophrenia must be linked to a speciation event (‘big-bang’ (Crow, 2008)) that influenced hemispheric specialisation and confer a balancing advantage to human populations. This factor, he argued, resided in the homologous regions of X/Y chromosomes and related to a key function influenced by cerebral asymmetry i.e., the faculty of language.

His views as to the exact genetic factor changed over time from viral progene to polymorphism, recombining to non-recombining regions of X/Y homologous class and later to epigenetics (Crow, 2010). Nonetheless, he was steadfast on the idea that schizophrenia results from an intrinsic genetic variation that determines our linguistic capacity. When arguing for the primacy of language in psychosis, Crow did not restrict himself to language-based cognitive tests or the measures of formal thought disorder (except in 2 of his works that were not pursued further (Ceccherini-Nelli & Crow, 2003; Ceccherini-Nelli et al, 2007)). Instead, he viewed all of schizophrenia’s major symptoms as disorders of language (Crow, 2004). To this, he repeatedly called upon de Saussure’s semiology, Buehler’s Sprachtheorie and Chomsky’s universal grammar. He argued for a hemispheric separation of the signifier (the referring word) and the signified (meaning of the referent) with bidirectional access being required during a discourse. When hemispheric balance is disrupted, as in schizophrenia, the use of a specific class of words called indexicals (e.g., I and you) which have interchangeable referents, become deviant. This disrupts the syntax of universal grammar. Self-other confusion typical of Schneiderian first rank symptoms ensue (Crow, 1998).

Today, many observations that diminish the premises of Crow’s arguments on asymmetry and evolutionary theory around language have come forth. His claims on the universality of incidence rates of schizophrenia have been shown to be inaccurate (McGrath et al., 2008). With respect to disrupted cerebral asymmetry, there is a lack of specificity to psychosis (Ocklenburg et al., 2024) and an absence of the sex effects that he anticipated (Schijven et al., 2023). No clear signal for positive genetic selection or balancing advantage has emerged; in fact, more recent studies indicate negative selection pressures (Pardiñas et al., 2018). There has been a consistent failure to implicate X/Y loci in extensive genetic studies of schizophrenia (Trubetskoy et al., 2022); instead, genetic evolutionary markers associated with schizophrenia have been reported at other loci not foreseen by Crow (Sandroni & Chaumette, 2025). Some observations are fatal to Crow’s assertions, but others call for new data (e.g., estimating the global incidence of linguistic dysfunction and Scheiderian symptoms in psychosis, epigenetic studies of sex chromosomes).

## 7 Crow’s approach to the schizophrenia question

Crow’s seminal works provide glimpses of his intellectual style and approach in psychiatric research (Table 1). He appreciated the necessity of having a guiding hypothesis that was parsimonious in its explanatory capacity (e.g., despite his initial proposal of subtypes, he continued to focus on a singular process that can explain the myriad of symptoms). Setting out to test such hypotheses, he followed a strong interdisciplinary approach. This pursuit was more than seeking collaborators from other fields of enquiry; he was thoroughly immersed in the relevant foundational arguments from the other disciplines that supported his parsimonious hypotheses. This depth of his scholarship, at times, led him to call for a revision of mainstream thought in other disciplines (e.g., his differences with Darwin’s and Chomsky’s notions of language evolution (Crow, 2004)). Some of these calls were in provocatively titled correspondences and reviews (e.g., see (Crow, 2004)). In the same inimitable style, he also engaged with contrasting views in the field (e.g. see (Crow, 2008) also see (Forti et al., 2015) and the response (Crow, 2015)). Importantly, he was prepared to change his own views if evidence necessitated a revision (e.g., rejected the viral contagion hypothesis on the basis of age of onset studies in siblings (Crow, 1984)).

He considered reliable clinical assessments to be critical to make progress with schizophrenia research and viewed some of the discrepancies in the field to be a result of variations in ascertainment of clinical features (e.g., his preference for simple, well-operationalised rating scales administered by clinically experienced investigators (Crow, 1985)).

To conclude, Crow not only raised key questions that continue to challenge contemporary psychiatric research, but also left behind a unique and integrative scientific approach to understand the biological complexity of psychosis. Despite the challenges faced by his theories, we expect his legacy to continue inspiring forthcoming enquiries in the neuroscience of schizophrenia.

## Figures and Tables

**Table 1:** Key aspects of Crow’s clinical academic approach in our view

Table 1: Key aspects of Crow’s approach
Anchoring efforts around a guiding hypothesis that was parsimonious in its explanatory capacity
Following a strong interdisciplinary approach
Immersing in the relevant foundational arguments from other disciplines
Engaging with contrasting views in the field
Changing views if evidence necessitated a revision
Emphasizing reliable clinical assessments
